# Supplementary material for: Cryo-EM structure of mammalian RNA polymerase II in complex with human RPAP2
Source: Commun Biol. 2021 May 21;4:606. doi: 10.1038/s42003-021-02088-z (PMC8140126; doi:10.1038/s42003-021-02088-z)
Supplement: Supplementary file 2 — Supplementary Information [file 42003_2021_2088_MOESM2_ESM.docx]

**Supplementary Information**

**Cryo-EM structure of mammalian RNA polymerase II in complex with human RPAP2**

Isaac Fianu^1^, Christian Dienemann^1^, Shintaro Aibara^1^, Sandra Schilbach^1^ and Patrick Cramer^1^*

^1^Max Planck Institute for Biophysical Chemistry, Department of Molecular Biology, Göttingen, Germany. *Correspondence: patrick.cramer@mpibpc.mpg.de

**
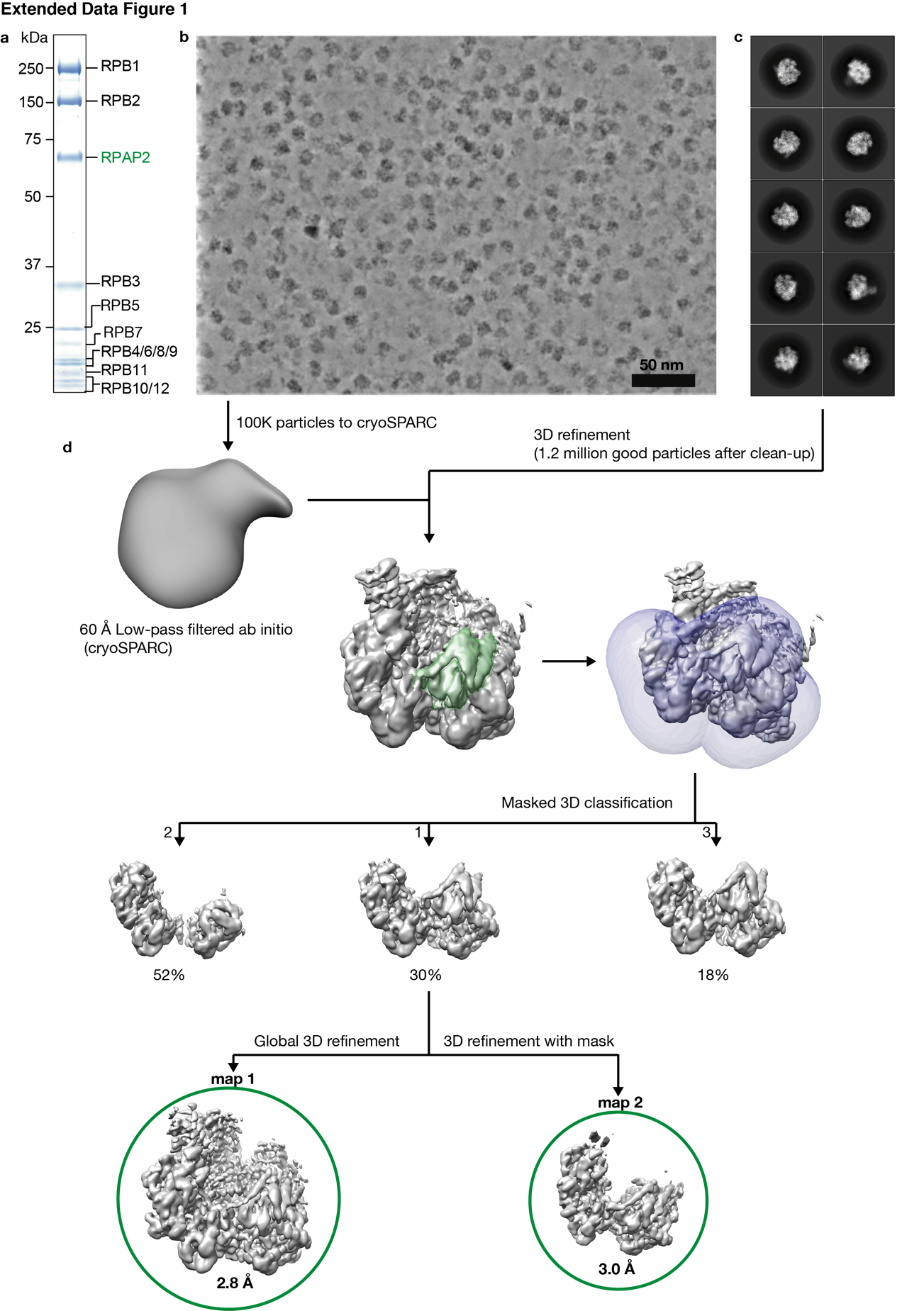
**

**Supplementary Figure 1 | Preparation of Pol II-RPAP2 complex and cryo-EM analysis.**

**a**, Quality of the Pol II-RPAP2 complex preparation. The peak fraction of the purified complex was analyzed by SDS-PAGE and stained with Coomassie blue.

**b**, Representative micrograph which was denoised using Warp. The scale bar has a length of 50 nm.

**c**, Representative 2D class averages.

**d**, Classification tree for data processing. A low-pass filtered ab initio 3D model generated using cryoSPARC. Clear extra density for RPAP2 is highlighted green. Mask region for focused 3D classification is shown as blue transparent surface. Names used to identify maps are shown above the corresponding maps. Map 1 is the global map and map 2 is the focused map.

**
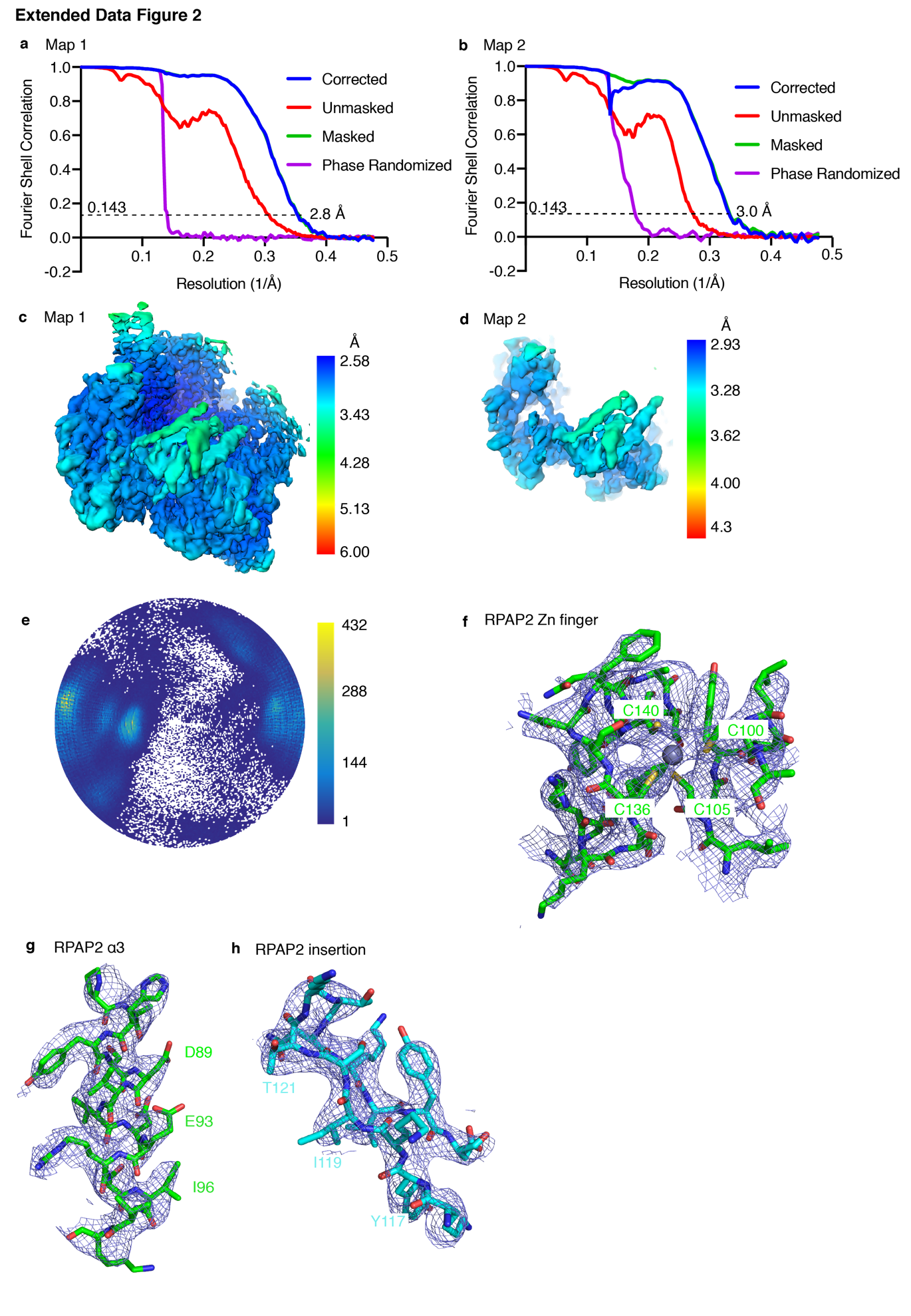
**

**Supplementary Figure 2 | Quality of cryo-EM data and density.**

**a-b**, Estimate of average resolution. The lines indicate the Fourier shell correlation (FSC) between the half maps of the reconstruction. FSC curves are shown for map 1 in **a** and map 2 in **b**. The cut-off of 0.143 is indicated by dashed lines as well as the estimated average resolution of each map.

**c-d**, Complete reconstruction colored by local resolution as implemented in RELION 3.0 for map 1 in **c** and map 2 in **d**. The scale bar shows the resolution (in Å) corresponding to each colored region.

**e**, Angular distribution of particles from overall refinement (map 1). Coloring from blue to yellow indicates the number of particles at a given orientation.

**f-g**. Carved cryo-EM density for selected RPAP2 elements (shown as sticks model). Cysteine residues coordinating the Zn atom (**f**), and residues in the interface with Pol II (**g** and **h**) are labeled.

**
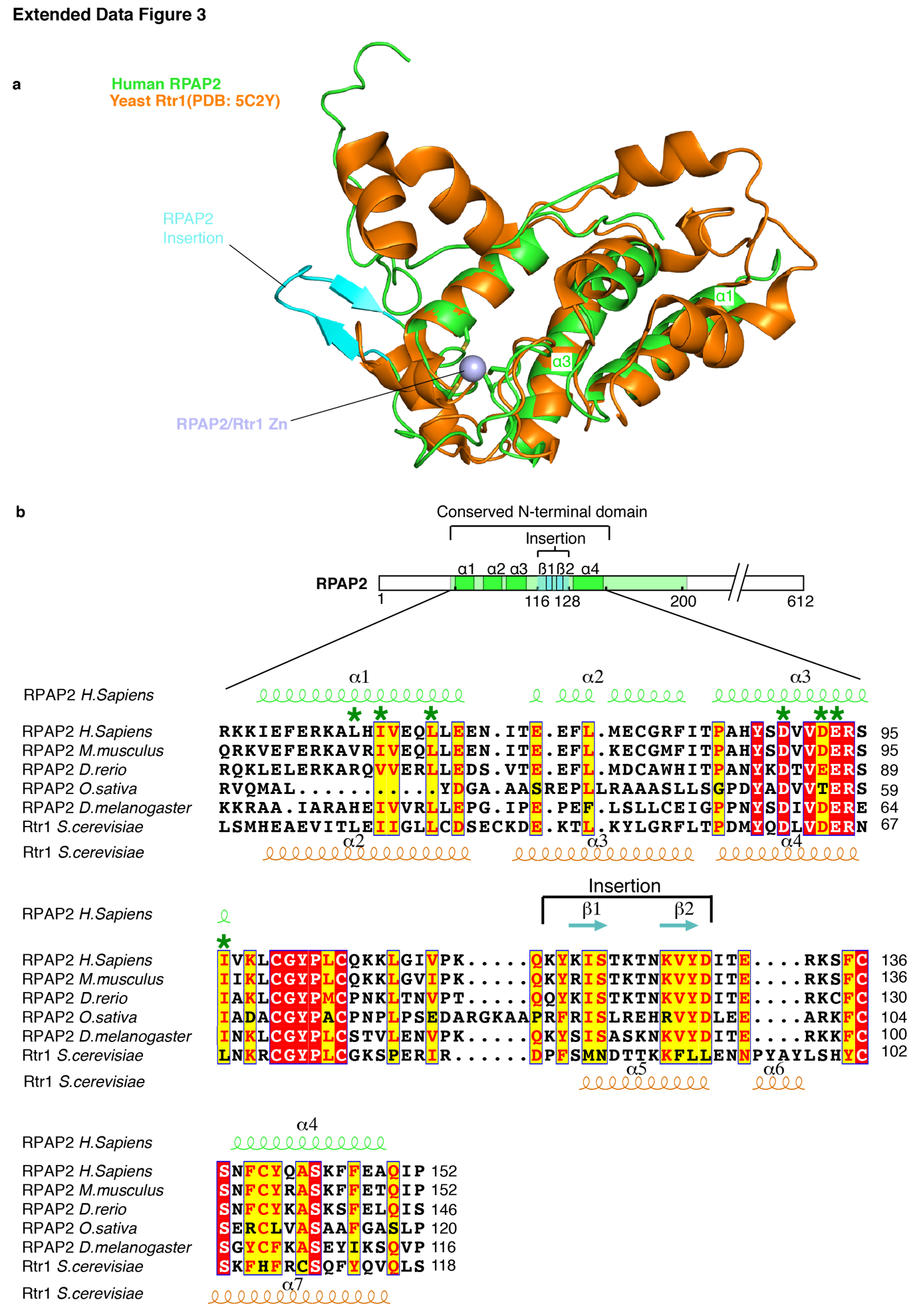
**

**Supplementary Figure 3 | Structure and sequence alignments.**

**a**, Comparison of the Pol II-bound human RPAP2 structure (green) with its free yeast homologue Rtr1 (orange, PDB: 5C2Y). Note the different structure of the insertion (cyan).

**b**, Multiple sequence alignment of RPAP2 conserved N-terminal domain. Secondary structure elements in our human RPAP2 structure are indicated on top and those of yeast Rtr1 (PDB: 5C2Y) are indicated below the alignment. Conserved residues are colored yellow and red and those at the interface with RPB5 are indicated with green asterisks. Note the different sequence and secondary structure of the insertion region for RPAP2 and Rtr1. Sequence alignments were performed with the T- coffee server[^33^](#_ENREF_33) and viewed with ESPript version 3.0[^34^](#_ENREF_34).

**
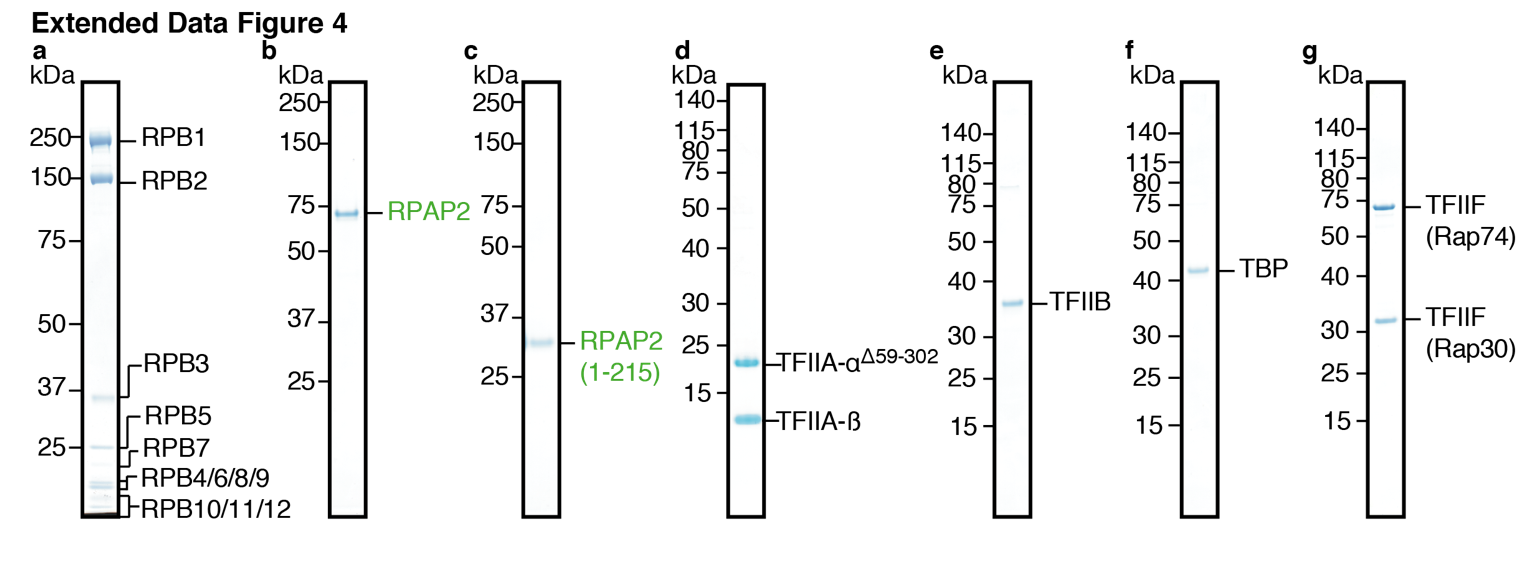
**

**Supplementary Figure 4 | Quality of proteins.**

Protein purification. A representative Coomassie stained SDS-PAGE of Pol II (**a**), RPAP2 (**b**), RPAP2(1-215) (**c**), TFIIA variant (**d**), TFIIB (**e**), TBP (**f**) and TFIIF (**h**).

**
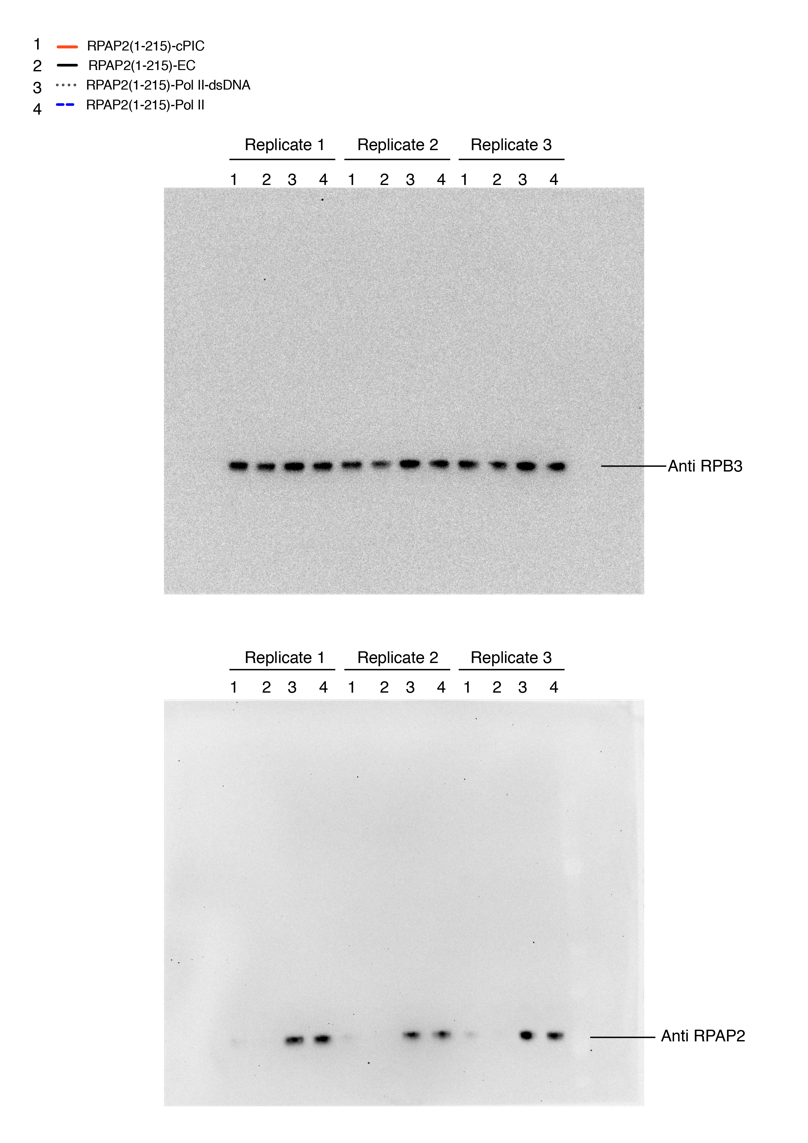
**

**Supplementary Figure 5 | Source data for Figure 2c.**

Top panel shows anti RBP3 (BETHYL #A303-771A) western blot analysis of peak fractions of competition assays and bottom panel shows anti RPAP2 (Thermo Fisher #PA5-61244) western blots of the same fractions. Results for three independent experiments are shown.

**Supplementary References**

33. Di Tommaso, P. et al. T-Coffee: A web server for the multiple sequence alignment of protein and RNA sequences using structural information and homology extension. in *Nucleic Acids Research* Vol. 39 13-17 (2011).

34. Robert, X. & Gouet, P. Deciphering key features in protein structures with the new ENDscript server. in *Nucleic Acids Research* Vol. 42 320-324 (2014).
